# Editorial: Fertility-preserving and fertility-sparing treatment approaches in gynecologic malignancies

**DOI:** 10.3389/fonc.2023.1199582

**Published:** 2023-06-02

**Authors:** Diego Raimondo, Antonio Raffone, Daniele Neola, Giuseppe Vizzielli, Donatella Caserta, Marcia Hall, Paolo Casadio, Renato Seracchioli, Lorenza Driul, Stefano Restaino

**Affiliations:** ^1^ Division of Gynaecology and Human Reproduction Physiopathology, Istituto di Ricovero e Cura a Carattere Scientifico (IRCCS) Azienda Ospedaliero-Universitaria di Bologna, Bologna, Italy; ^2^ Department of Medical and Surgical Sciences (DIMEC), University of Bologna, Bologna, Italy; ^3^ Department of Neuroscience, Reproductive Sciences and Dentistry, School of Medicine, University of Naples Federico II, Naples, Italy; ^4^ Department of Medicinal Area (DAME) Clinic of Obstetrics and Gynecology, Santa Maria della Misericordia Hospital, Azienda Sanitaria Universitaria Friuli Centrale, Udine, Italy; ^5^ Department of Medical and Surgical Sciences and Translational Medicine, Sapienza University of Rome, Sant’Andrea University Hospital, Rome, Italy; ^6^ Department of Medical Oncology, Mount Vernon Cancer Centre, Northwood, United Kingdom; ^7^ Department of Medicine, University of Udine, Udine, Italy

**Keywords:** cancer, tumor, oncofertility, reproduction, offspring

The advances in the diagnosis of cancer made gynecologic malignancies more and more frequent, with an estimated incidence of 222,700 new cases in Europe (1).

The concomitant recent increase of the age of first pregnancy in industrialized countries resulted in a considerable number of diagnosis of gynecologic malignancy in women of childbearing age (2). The diagnosis of a gynecologic malignancy can affect the fertility capability of a woman both from an anatomical point of view (the primary approach to a gynecologic malignancy is to surgically remove the affected organ) and from the gonadotoxicity derived from medical or radiation treatment (3).

In most cases, these women have not completed their wish of maternity and are very motivated to preserve fertility. In fact, recent studies have shown that the potential iatrogenic loss of fertility has a profound impact on young women and it may be more stressful than the cancer diagnosis itself (4, 5). Thus, preserving the ability to have biological offspring is the most important goal for many survivors of cancer (6).

In this scenario, the preservation of fertility in women with gynecological malignancies is an important issue and must be taken into consideration, in order to guarantee a patient-tailored approach, which is paramount in modern oncology. However, this is not always possible, since fertility-sparing strategies are feasible only in early stage diseases. On the other hand, advanced diseases usually require a radical approach leading to irreversible infertility, even if some authors hypothesised the futuristic use of uterus transplantation after an eventual previous oocyte cryopreservation (7).

The aim of this Research Topic was to focus on novel frontiers in the fertility-sparing treatment (FST) of gynecologic malignancies, with particular attention on endometrial carcinoma (EC), which is without doubt the most frequent among gynecologic tumors, and the one for which more advances in preservation of fertility were made (8).

Nowadays, inclusion criteria for fertility-sparing treatment of endometrial carcinoma are: women younger than 40 years who plan to conceive as soon as possible after remission, histology of grade 1 EC, endometrioid histotype, International Federation of Gynecology and Obstetrics (FIGO) stage IA with neither myometrial nor adnexal involvement, negative lymphovascular space invasion (LVSI) (9). However, the ESGO/ESHRE/ESGE Guidelines (10) have introduced the possibility to consider FST also in grade 2, endometrioid histotype, EC and in case of initial infiltration of the myometrium (1-2 mm) (8). In this sense, Ronsini et al. provided a systematic review of the literature collect the most incisive studies about the possibility of conservative management for patients with grade 2, stage IA EC.

Obesity is a known risk factor for EC, however, it is also a risk factor for FST failure (11). Chen et al. evaluated the effectiveness and prognosis of FST on EC and atypical endometrial hyperplasia (AEH) in patients with BMI ≥ 30, finding that a weight loss of more than 10% may have a positive influence on response, recurrence and pregnancy rates. In addition, they found that FST with a GnRH agonist was more effective compared to progestin therapy, probably due to less effect on weight gain.

In fact, obesity, metabolic syndrome and insulin resistance seem to have a strict relationship with EC and, as a consequence, with the success of a FST approach. Risk factors for recurrence and complete remission time after recurrence (RCR time) of EC and Atypical Endometrial Hyperplasia (AEH) were investigated in Li et al. study. Interestingly, both insulin resistance and metabolic syndrome were significantly associated with recurrence and longer RCR time in AEH and early EC.

The prevention of a relapse is the main issue in oncology and He et al. proposed some maintenance therapy regimes with low-dose oral progesterone, levonorgestrel intrauterine device (LNG-IUD) and combination oral contraceptive (COC) to prevent a recurrence after FST of EC. The recurrence rate of AEH and EC in the maintenance therapy group resulted significantly lower than those in the non-maintenance group, highlighting this regimen as a potential option to prevent recurrence and also to protect the endometrium in patients treated with assisted reproductive technology (ART), greatly reducing the recurrence rate after ART.

In the context of FST of EC, Khan et al. analyzed the effectiveness *in vitro* of Guizhi Fuling Wan, a traditional Chinese medicine which is used to reduce recurrence of endometriosis, improve pregnancy outcomes and treat uterine fibroids. In this study, Guizhi Fuling Wan seems to inhibit invasiveness in both high and low grades of EC cells, at doses estimated to be comparable to those being used in clinical testing and traditional practice.

Ovarian carcinoma is, in general, more frequent in postmenopausal women; however, it can also occur in young premenopausal women (12). In this case, a fertility-sparing approach requires unilateral removal of adnexa, preserving the uterus and contralateral ovary, followed by 3–6 cycles of platinum-based adjuvant chemotherapy. However, platinum-based chemotherapy can induce gonadotoxicity and affect ovarian function. Xie et al. assessed the effectiveness of gonadotropin-releasing hormone (GnRH) agonist co-therapy for the preservation of ovarian function in patients with ovarian malignancy who underwent unilateral salpingo-oophorectomy and platinum-based chemotherapy, demonstrating a protective role of GnRH agonist uring platinum-based adjuvant chemotherapy in young patients with ovarian malignancy.

Lastly, Xu et al. reported a case of FST in a patient with a gestational trophoblastic neoplasia (GTN), in particular an invasive mole treated with methotrexate chemical therapy, uterine artery embolization simultaneously, and with a cycle of chemotherapy with etoposide, methotrexate, dactinomycin, cyclophosphamide, and vincristine (EMACO). This report provided a valuable demonstration of the potential feasibility and effectiveness of conservative treatment for fertility preservation in such scenarios.

We hope this Research Topic will provide new and personalized strategies for the fertility-sparing treatment of gynecological malignant tumors to reduce the adverse consequences of gynecological malignancies in women and guarantee a more tailored approach to this type of tumors.

## Author contributions

DR, DC, MH and SR reviewed the articles included in the research topic. AR, DN and GV worked on the manuscript preparation. PC, RS and LD supervised the whole process and resolved in case of doubts. All authors contributed to the article and approved the submitted version.
